# Supplementary figures and images for: DNA repair function scores for 2172 variants in the BRCA1 amino-terminus
Source: PLoS Genet. 2023 Aug 14;19(8):e1010739. doi: 10.1371/journal.pgen.1010739 (PMC10449183; doi:10.1371/journal.pgen.1010739)

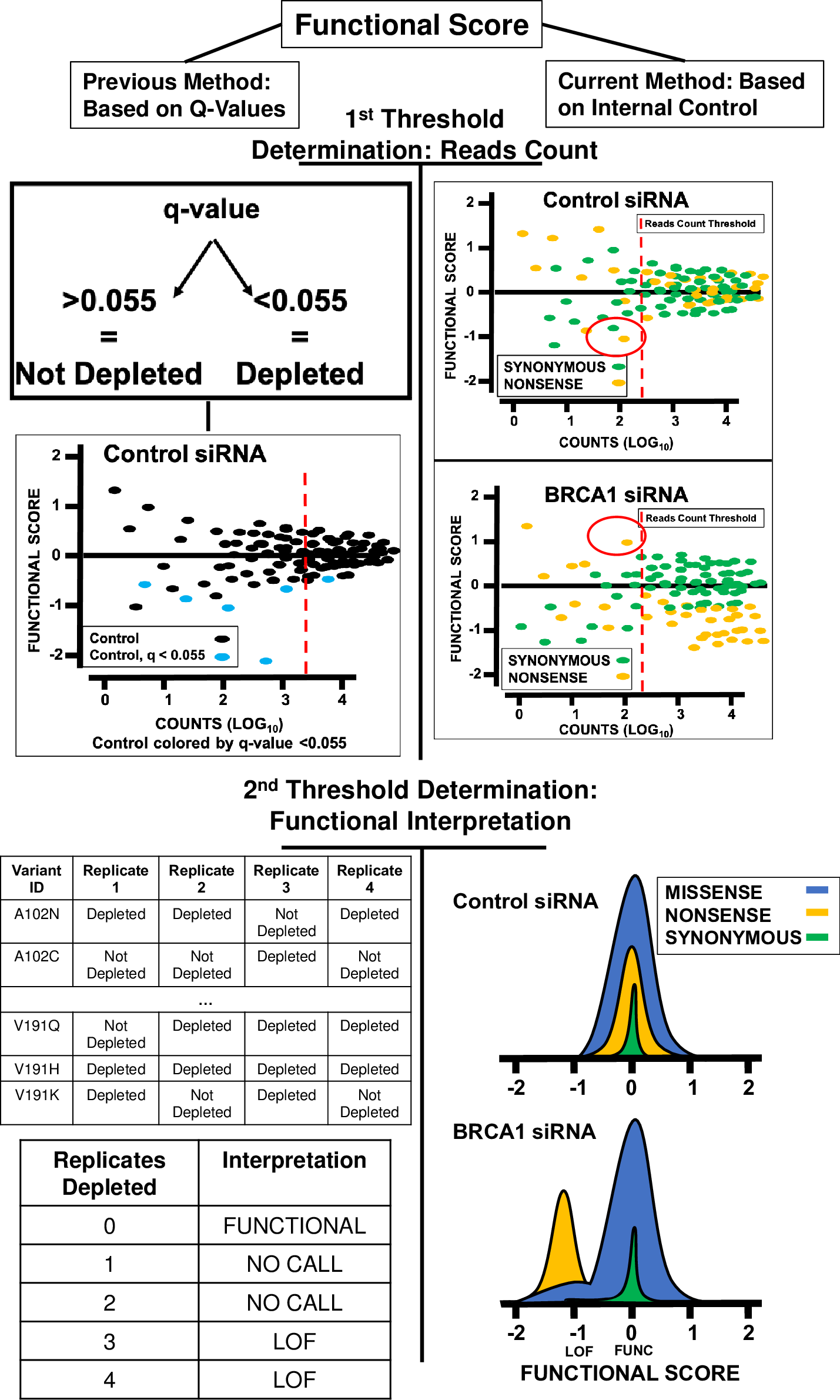

Supplement: S1 Fig — The left side of the figure shows the original analysis steps used in the paper published in 2018 [17], and the right side of the figure summarizes the steps changed in the current approach. The analytic pipeline previously described used a binary classifier based on the false-discovery rate (q-value) as a quantifier. The binary classifier was created by designating variants with a q value <0.055 as ’depleted’ and variants with a q value > 0.055 as ’not depleted.’ The overall depletion score was calculated by counting the number of times a variant was depleted across the four replicates. In the current study, performance was optimized using internal controls (synonymous and nonsense variants) in cells containing endogenous BRCA1 (control siRNA) and in cells with the endogenous gene silenced (BRCA1 siRNA). We evaluated the read counts (horizontal axis) and at low read counts the datapoints deviated from normal function (0 on the vertical axis) in control cells and in the BRCA1 siRNA transfected cells, synonymous variants deviated from normal function at low read counts. The red circles indicate data for variants which deviate from expectations and were used to establish the read-count threshold. This analysis set the minimum number of reads required for a variant to be included in the analysis. After establishing the read-count threshold, the threshold for functional versus LOF was determined. In the previously published analysis, if the q-value for a variant indicated depleted in three or four replicate experiments, then the variant was considered LOF. If the q-value indicated zero replicates depleted, then it was interpreted as functional. If a variant was depleted in one or two replicates, then no functional determination was made. In the current analysis, the population distributions of missense, nonsense, and synonymous, shown here as expected distributions, were used to determine the threshold for functional interpretation. The cut-off values were established b [file pgen.1010739.s001.tif]

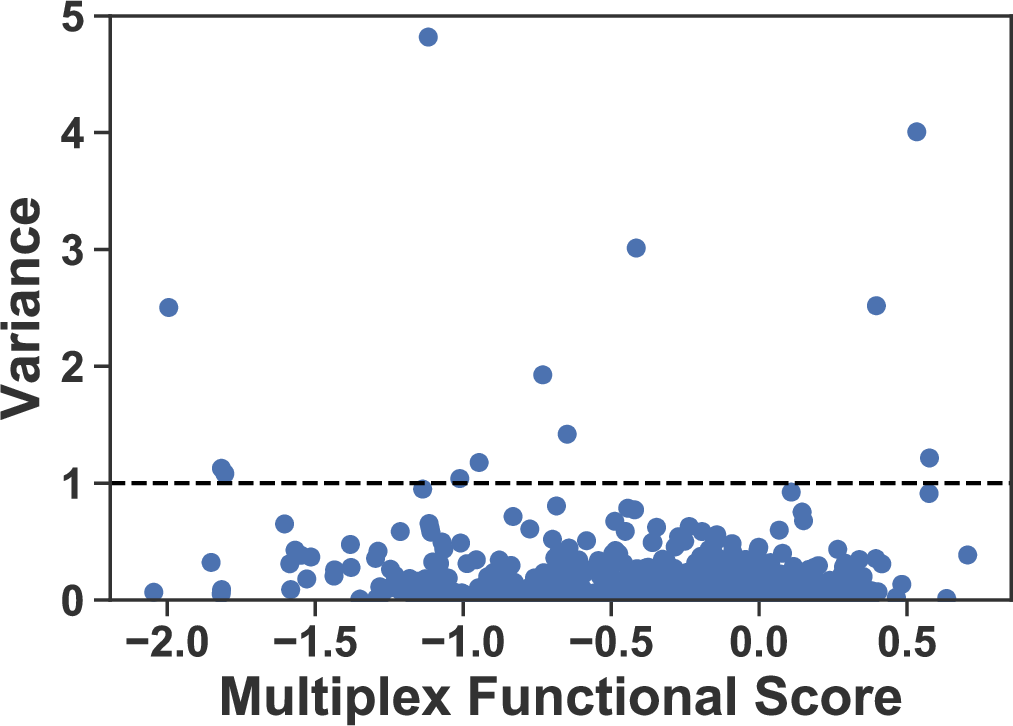

Supplement: S2 Fig — The DNA repair functional score variability of BRCA1 variants was evaluated by plotting the standard variance across four replicates (y-axis) against the mean functional score (x-axis). Variants with a standard deviation greater than 1 were removed from further analysis. (TIF) [file pgen.1010739.s002.tif]

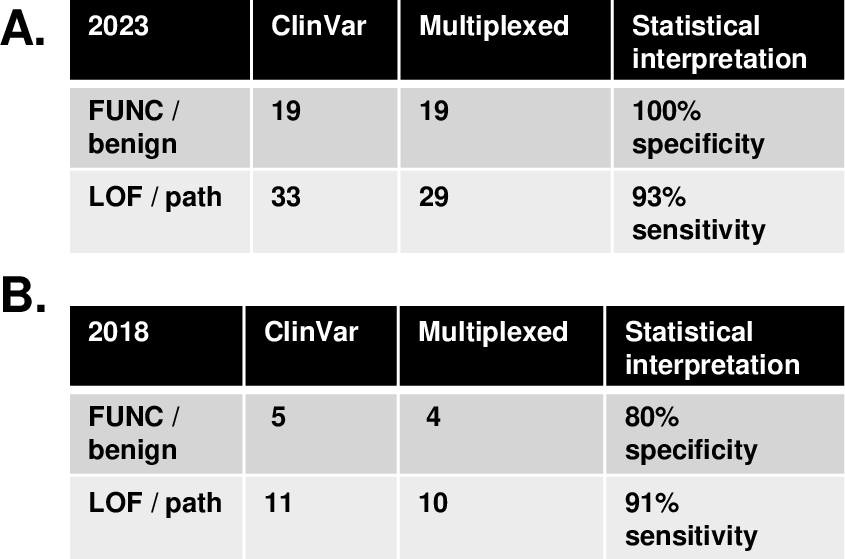

Supplement: S3 Fig — A. The current analysis of the multiplexed HDR assay was compared with variants with known clinical impact listed in ClinVar. B. The functional determinations using the previously published analysis was compared with variants with known clinical impact listed in ClinVar. Due to updates in the ClinVar database, the number of variants shown in this table is different from originally published. (TIF) [file pgen.1010739.s003.tif]

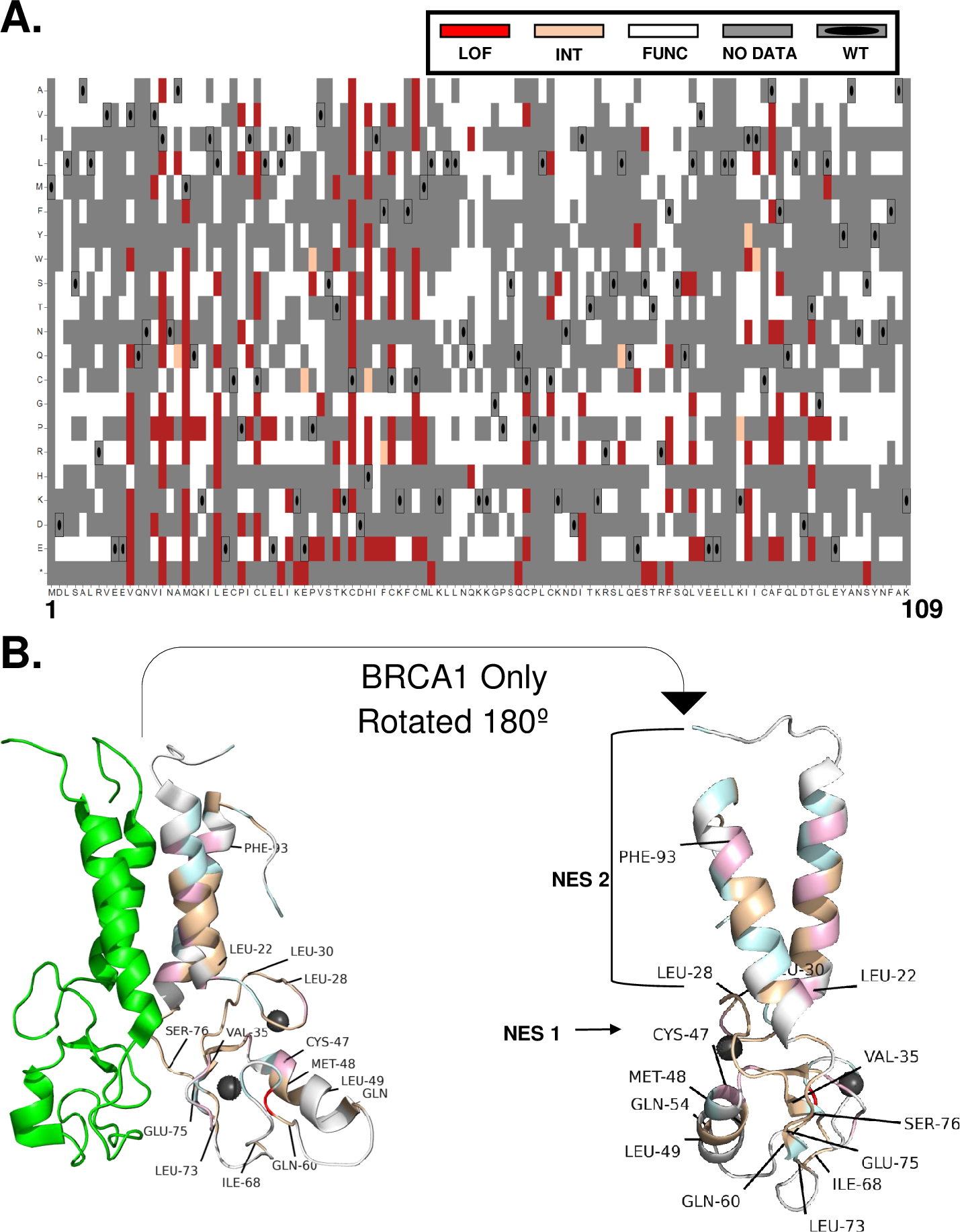

Supplement: S4 Fig — A. The close-up view of the sequence-function map from Fig 7 shows the relationship between the sequence of the RING domain (positions 1–110 of BRCA1) and the functional impacts of each variant tested. The color coding of the variants represents the functional performance of the RING domain: red for loss of function, white for functionally normal, peach for intermediate function, and gray for variants with no determinations (read-counts below the threshold for inclusion or variant not detected). The x-axis represents the wild-type amino acid one-letter code, and the y-axis represents the mutated amino acid one-letter code. B. This visualization shows the interaction between BRCA1 and BARD1 proteins (PYMOL:1JM7), with BRCA1 residues colored based on their performance in the functional assay. Red represented loss of function in all substitutions, light pink represented more than half of substitutions resulting in loss of function, peach for less than half of substitutions resulting in loss of function, and white for maintenance of function in all tested substitutions. The zinc atoms in the RING zinc-finger are colored grey. BARD1 peptide was colored green. In the close-up view of the alpha-helices of BRCA1, the nuclear export sequences are indicated with brackets and arrows, and the helices have been rotated to show the face of BRCA1 that interacts directly with BARD1. (TIF) [file pgen.1010739.s004.tif]
