# Supplementary material for: DNA repair function scores for 2172 variants in the BRCA1 amino-terminus
Source: PLoS Genet. 2023 Aug 14;19(8):e1010739. doi: 10.1371/journal.pgen.1010739 (PMC10449183; doi:10.1371/journal.pgen.1010739)
Supplement: Attachment — Submitted filename: BRCA1-N Response letterJune 9 2023.docx [file pgen.1010739.s014.docx]

Reviewer's Responses to Questions

**Comments to the Authors:
Please note here if the review is uploaded as an attachment.**

Reviewer #1: BRCA1 is a breast and ovarian cancer-specific tumor suppressor protein, and its homology-directed repair (HDR) activity is known to be important for its function as a tumor suppressor. Therefore, evaluating BRCA1 variants' function is critical for accurately diagnosing hereditary cancer. The authors previously published the results of a multiplexed HDR assay for 1056 amino acid (aa) substitutions in the amino-terminal region of BRCA1, 2-192 aa. In this study, the authors improved the analysis pipeline. They re-analyzed the variants in 2-192 aa and added the results of the new variants in 193-302 aa of BRCA1. Thus, they analyzed 2172 variants and improved the sensitivity and specificity for this assay compared with the determination by ClinVar, which considers clinical information.
This study is novel and interesting and will contribute directly to evaluating BRCA1 variants. Furthermore, this strategy will provide valuable insight into diagnoses of other hereditary cancers. However, some points below should be revised before the publication in PLOS Genetics.
We thank the reviewer for their thoughtful comments, and we have made suggested changes that improve the presentation of the manuscript.

General Comments:
1. Distinguishing pathogenic variants from benign variants is important. However, some missense variants cause slight protein conformation change that decreases the HDR activity intermediately and increases the cancer risk moderately. The authors should discuss this in the sections of the Discussion.

We now address the issue of variants with intermediate functional scores on lines 320-323, in the Discussion section.

2. This study will also contribute to predicting the tumor sensitivity to PARP inhibitors and other DNA-damaging agents. The authors should comment on this in the Introduction and/or Discussion sections.

To address this concern, we have added comments on lines 63-64 (Introduction) and on lines 243-246 and added Figure 6C,D (Results) to compare our results to those of another repair assay and to resistance to a PARP inhibitor.

Specific Comments:
1. In Line 227, a report of the functional assay (Endo et al. Cancer Res Commun. 2021 1(2):90-105) should be cited as a singleton assay.

We decided to include in the Figures a comparison of the current multiplexed results to the DNA repair assay and the Olaparib resistance assay in Endo et al. These are now Figures 6C and 6D and text is on lines 243-246.

2. In Line 249, reference number 38 seems to be a mistake.

We have replaced the reference with the correct publication (now ref 36 and cited on line 257). Thank you for spotting that error.

3. In Figure 6B, indicating the four variants in the graph are better.

We have colored the discordant variants red in the figure.

Reviewer #2: In this study, the authors have functionally characterized BRCA1 variants of uncertain significance (VUS) using a previously described GFP-based homology-directed DNA repair assay. The authors have focused on residues 2-302 of BRCA1, which includes the RING domain that binds to BARD1. The experimental approach involves changing each codon to all possible other codons using a pool of BRCA1 cDNA with an NNK substitution at each codon. As a result of this substitution, each amino acid is changed to all possible 19 non-WT residues. The authors have used a new analysis approach to reanalyze previously published results for BRCA1 residues 2-192. They have modified the method to assess whether the data on the abundance of a variant is sufficient to be included in the analysis without resulting in erroneous classifications of variants. The new approach also differs in how nonfunctional and functional variant are classified. The new approach resulted in an increase in the number of variants that could be classified. In addition, the new approach resulted in increased sensitivity and specificity. In addition, they have generated new variants and determined functional scores for 808 additional variants spanning residues 193-302.

We thank this reviewer for their careful consideration of this manuscript.

Overall, the study has resulted in functional classification 2172 BRCA1 variants from the N-terminal region of the protein. While the findings are important and relevant, a major concern is the lack of any information on the nucleotide change that resulted in a given amino acid change. This is especially important when comparing their variant classification with ClinVar and SGE data. It is unclear if the authors compared the correct variants (i.e., correct nucleotide change) or compared any variant that resulted in the same amino acid change indicated in ClinVar. It is critical that the authors clarify this. Although in the present study, BRCA1 variants were expressed using a cDNA construct, it is known how silent variants can result in a non-functional protein but affecting splicing. It is equally important that when calculating specificity and sensitivity, variants that have identical nucleotide change should be compared and not identical amino acid change. Authors must include a “nucleotide change” column following HGVS nomenclature system, in Supplementary Tables S1-S5.

We have added a new Supplemental table (Table S9) that includes the single nucleotide changes, but this is a complex issue. Because we mutated the sequence of the BRCA1 protein using a cDNA expression vector, we have in some cases multiple nucleotide changes that were combined when evaluating an amino acid change. We do not want to suggest that the nucleotide changes in the cDNA expression vector in any way reflect the nucleotide changes (usually in a SNV) in the endogenous genomic location. We add text to describe this issue in the Methods (lines 417-421).

Minor comments:
1. Authors have compared their results with SGE data. A number of other CRISPR-based high throughput functional assays have been used to examine the variants in the N-terminal region of BRCA1. Authors should correlate their functional results with these functional assays (e.g., Kweon et al., 2020, Hanna et al, 2021, Sangree et al., 2022)

Those three studies did not focus on BRCA1 as was done in the current study, and they each only analyzed a very small set of variants in BRCA1, and there was no overlap with the variants in the results of this manuscript. In the RING domain, Sangree examined L63P, C64R, P34F; Hanna examined C64Y, L52F, and D67N; and Kweon examined L52F, and none of these variants were captured in the assay for the current study. For this reason, a direct comparison of our results could not be made to those papers. We did not feel we should include in the text of the manuscript a discussion about the small number of variants in the amino terminus of BRCA1 in those papers with high throughput analyses.

2. Figure 4, comparing the functional scores of BRCA1 variants in the presence (siControl) and absence (siBRCA1) is an excellent idea. The results are overall very convincing and demonstrate the suitability of the functional assay for classification of BRCA1 VUS. It is unclear why some pathogenic variants behave as benign. Are these synonymous variants that may affect splicing? This effect may be masked because of the use of a cDNA construct.

We discuss our ideas on why some pathogenic variants functioned normally. For the most part, these correlate with variants that affect splicing. One of the pathogenic variants could not be explained by splicing effects, and its functional score was shifted to the left, but it did not shift far enough to be classified as LOF. (lines 191-196).

3. In the text, when mentioning the number of variants that were generated and analyzed in the present study, the authors should mention the total number of variants that were expected to be generated. It is evident from Figure 7 that the number of variants that were generated in the new set was very low. Can the authors comment on why in spite of refining their analysis strategy, the recovery appears to be very low.

Compared to pools 1 and 2, pool 3 library generation was lower, which yields a lower number of variants available to assess. Furthermore, this number of available of nonsense and synonymous variants in pool 3, directly affecting the cutoffs applied. Even so, 413 variants were tested. Though the eye is attracted to the gaps, with a possible 2200 variants in pool 3, obtaining functional results on 413 variants is still a high number.

4. Figure S3: In 2018, one benign variant was not classified correctly as a benign variant. However, in the present study, all 19 benign variants were correctly classified as a result the specificity went up to 100%. Was this because of the change in the cut-off scores used for considering a variant to be functional or non-functional?

We clarified this important point in the text (lines 205 to 209). In the 2018 paper, there were five benign variants analyzed, and four were scored as functional (zero times depleted). The other variant (Y179C) was depleted one time and was not functionally classified. In the new analysis protocol, we captured those five variants plus 14 more and all were scored as functionally normal.

Reviewer #3: The Findlay et al publication of this MAVE was a landmark, presenting evidence of functional impact in 1056 amino acid substitutions in BRCA1. This manuscript presents a new analysis of the MAVE data with a more sensitive methodology, in which the authors can now make function calls for as many as 2172 variants. These new results are largely concordant with the previous ones, and where they differ, the new results seem to be more accurate. This manuscript stands to become a highly impactful publication, with a few modifications.

Thank you for the careful consideration of our study. Additionally, we have uploaded our results to the MaveDB database, the accession number is pending.

Major comments:
The comparison to ClinVar does not exclude variants which have been interpreted using functional data. Given the impact of the Findlay et al publication, it's highly likely that some of the new ClinVar classifications were made in part with the data from this very assay. At very least, such classifications should be excluded from the comparison. An even better approach would be to exclude all classifications that were made on the basis of functional evidence, since even a different functional assay could introduce circularity.

For the ClinVar comparison, we only used the clinical classifications and not the functional data. In essence, we compare to the functional data in Figure 5, and we do not double count the variants as part of the ClinVar analysis. We clarify this point in the results (line 184-185).

Minor comments:
Line 110: "removed the variants for which the abundance of variants...". This is awkward wording, using "variants" twice in the same phrase.

This sentence was reworded on line 111.

Line 148: "all variants" is confusing, as it suggests "each variant". Perhaps it would be better to refer to the overall variant distribution?

This sentence was reworded on line 149; we changed “all variants” to “the total population”.

Lines 322-326: Awkward wording. This sentence is difficult to parse.

We deleted the sentence.

Figure S1: There are three variants circled in red at the right, but the caption does not indicate their significance.

The caption was altered to include an explanation of the circle.

Figure S4: In this image, it's difficult to distinguish between red (LOF: all substitutions) and magenta (LOF: most substitutions)

We altered the color scheme and hope it is easier to discern the differences.

Finally, while the data is available in GEO, I urge the authors to make these data available also on MaveDB.

We have deposited the dataset to MaveDB, and we cite this in the revised text. (line 425)
