# Supplementary material for: DNA repair function scores for 2172 variants in the BRCA1 amino-terminus
Source: PLoS Genet. 2023 Aug 14;19(8):e1010739. doi: 10.1371/journal.pgen.1010739 (PMC10449183; doi:10.1371/journal.pgen.1010739)
Supplement: Attachment — Submitted filename: Response letter July 13.docx [file pgen.1010739.s015.docx]

We thank the reviewers for their consideration of this study. The review process has greatly improved the presentation of the manuscript.

**Comments to the Authors:
Please note here if the review is uploaded as an attachment.**

Reviewer #1: The revised manuscript significantly improved, and the authors adequately addressed my previous concerns.

Reviewer #2: The authors have satisfactorily addressed my concerns.

Reviewer #3: This manuscript is a strong, impactful piece of work. The authors have addressed most of the reviewer comments. This reviewer remains concerned that the comparison of the functional assay results to the clinical classifications in ClinVar might be inflated, because some classifications might be informed by functional data, and in particular by the results from the Findlay et al publication. But given that many ClinVar classifications aren't explicit about what information informed the classification, I accept this as a limitation. I suggest that the authors add a brief disclaimer statement saying that it's possible that some ClinVar classifications could've been informed by the earlier publication on this assay.

We added this caveat as requested by the reviewer. It is in lines 199-203.
